# Cisplatin-associated ototoxicity: perspectives from a single institution cervical cancer cohort and implications for developing a locally responsive monitoring programme in a public healthcare setting

**DOI:** 10.1186/s12913-022-08099-8

**Published:** 2022-06-18

**Authors:** Jessica Paken, Cyril D. Govender, Mershen Pillay, Vikash Sewram

**Affiliations:** 1 Discipline of Audiology, School of Health Sciences, University of KwaZulu-Natal, Private Bag X54001, Durban, 4000 South Africa; 2 Speech and Language Therapy, Massey University, Albany (Oteha Rohe) Campus, Building 84, Auckland, New Zealand; 3 African Cancer Institute, Department of Global Health, Faculty of Medicine and Health Sciences, Stellenbosch University, P.O. Box 241, Cape Town, 8000 South Africa

**Keywords:** Audiological assessment, Ototoxicity, Monitoring programme, Cisplatin, Cervical cancer, South Africa, Hearing loss

## Abstract

**Background:**

Ototoxicity monitoring is uncommon in South Africa, despite the increased use of ototoxic medication to manage the burden of disease in the country. The successful implementation of such a protocol requires cognisance of contextual realities and multiple dimensions for consideration from both patients and service providers. As part of an ongoing cohort study on cisplatin-associated ototoxicity and efforts to better inform the implementation of such programmes, the perspectives of cervical cancer patients and healthcare workers towards ototoxicity monitoring were assessed.

**Methods:**

This concurrent-triangulation mixed-methods study was conducted at a tertiary hospital in South Africa. Self-reported questionnaires from patients (*n* = 80) and healthcare personnel comprising clinicians, oncology nurses, pharmacists, and radiotherapists (*n* = 32), results of audiological evaluations, researcher field notes, and estimated patient and service provider costs contributed to data for this study. Data analysis included descriptive statistics, comparison of test characteristics and deductive thematic analysis.

**Results:**

The ototoxicity monitoring programme was positively received by the participants, with 90.6% of healthcare personnel and 89% of patients reporting it to be beneficial. The clinicians (76.6%) were identified as the main providers of information on the effects of chemotherapy medication and made the necessary referrals for audiological evaluation. The approximate cost of setting up such a programme included purchase of equipment (US56 700) and the appointment of an audiologist (US 26 250). The approximate costs to patients included transport costs (US$ 38) and the loss of income for the day (US 60), calculated at the minimum wage per hour, if employed. Creative appointment scheduling, easy facility access and detailed locally comprehensible couselling improved patient compliance to the programme. Whilst the sequential use of American Speech-Language-Hearing Association (ASHA) and National Cancer Institute Common Terminology Criteria for Adverse Events (NCI-CTCAE) criteria aided in an evidence-informed approach to aural rehabilitation, DPOAEs and speech discrimination displayed low sensitivity (range 1.45% – 22.39%) but high specificity (range 77.78% – 100%) when identifying ototoxic change.

**Conclusion:**

This novel study, through a ‘real-world’ experience, has revealed that an ototoxicity monitoring programme is feasible in South Africa, through meaningful engagements with- and considerations from- patients and service providers regarding planning, delineation of responsibilities and cost implications. The findings can potentially serve as a roadmap for other limited resource environments.

**Supplementary Information:**

The online version contains supplementary material available at 10.1186/s12913-022-08099-8.

## Introduction

More than 5% of the world's population (approximately 432 million adults) currently has a disabling hearing loss, with this estimation likely to increase to 10% (over 700 million) by 2050 [1]. Some of the more significant contributors to the increasing global estimates for disabling hearing impairment are longer lifespans, greater exposure to recreational and occupational noise, and ototoxicity [2]. Ototoxicity is defined as the chemical injury to the structures and functioning of the auditory-vestibular system occurring as a side effect of pharmacotherapy and exposure to chemicals and ionizing radiation [3]. With over 600 drugs considered to be ototoxic, the most commonly used include the platinum-based chemotherapeutic agents (cisplatin and carboplatin), aminoglycoside antibiotics, loop diuretics, macrolide antibiotics, antimalarials [4], and antiretroviral medication [5].

Cisplatin, known as the "Penicillin of cancer" [6], is used in the treatment of many cancers [7] but is also notorious for its ototoxic properties, with the reported prevalence varying between 50–80% and 60–90% in adults and children, respectively [8]. It is well documented that hearing loss, and especially an untreated one, can adversely impact the quality of life of affected individuals [9, 10], but is more overwhelming at a time when their critical healthcare decisions are discussed [11], and significant others rally together to provide support [12]. Therefore, early detection of hearing loss and proactive management and care through an ototoxicity monitoring programme is essential [13].

Ototoxicity monitoring in South Africa should be an integral component within the audiologist's scope of practice, especially given the country's burden of disease profile within the communicable (Human Immunodeficiency Virus, Multi-Drug Resistant Tuberculosis), and non-communicable (Cancer, Diabetes, Hypertension) disease envelope. Despite international guidelines for ototoxicity monitoring [14] being published almost 26 years ago, the Speech-Language and Hearing Professions Board of the Health Professions Council of South Africa (HPCSA) only released its guidelines in 2018 [15]. Ototoxicity monitoring, with nationally accepted guidelines, is yet to become part of mainstream practice, globally [16]. By way of examples, in New Zealand, it was found that that there was no nationally accepted ototoxicity monitoring programme and the need for closer collaboration between oncologists and audiologists were identified [17]. Oncologists and infectious disease physicians in the USA were also found not to be fully aware of the diversity of an audiologist’s practice, potentially influencing the success of an ototoxicity monitoring programme [12]. Similar findings were evident in the UK, even amongst audiologists (71%) who were not aware of an ototoxicity monitoring programme within their services and baseline assessments only being conducted following patient's auditory complaints [18]. A survey in South Africa revealed a need for oncologist’s education on ototoxicity and its importance on the survivor's quality of life [19]. A more recent study revealed that healthcare personnel, managing cancer patients, need to improve their awareness of ototoxicity, with audiologists requiring greater awareness of monitoring programmes [20]. Similar sentiments were expressed by Khoza-Shangase and Masondo [21], who also added that audiologist’s training in ototoxicity monitoring, i.e., assessment and management, has not translated well to their practices within the South African context.

The national guidelines for South Africa, issued by the HPCSA in 2018 [15], and based primarily on American Speech-Language-Hearing Association (ASHA) [14] and American Academy of Audiology (AAA) [22] protocols represents a well-structured approach for monitoring ototoxicity. However, human resource and infrastructure constraints [23] hamper the efficient rollout of this programme and therefore, a more locally responsive programme cognisant of the contextual realities of the environment [21] is paramount to successful implementation. Furthermore, there has been no formally implemented ototoxicity monitoring programme to identify and monitor ototoxicity in patients receiving cancer chemotherapy, despite global projections reporting a 47% increase by 2040 [24]. It is also estimated that by 2040, the incidence of cancer in South Africa will increase by 60.8% with cancer of the cervix (disease with which the current patient cohort is defined) projected to increase by 48% [25].

This investigation, which adopted a concurrent triangulation mixed-methods approach, formed part of an ongoing cohort study on cisplatin-associated ototoxicity. The aim was to assess the perspectives of cervical cancer patients and their healthcare team towards ototoxicity monitoring, with a view to further informing feasibility, implementation and integration of an ototoxicity monitoring programme into the clinical environment.

## Methods

### Study site and population

The study was conducted at a tertiary level referral hospital in KwaZulu-Natal, South Africa.

This site was selected due to its large referral base, providing highly specialized healthcare to the Western half of the province. Being one of the main referral centres for cancer patients and having an Audiology department deemed this site most suitable to achieve the aim of this study.

The study participants included:

- 80 females diagnosed with incident cervical cancer, and
- 32 healthcare personnel [six clinicians (oncologists and registrars) working within the oncology department, eight oncology nurses, nine pharmacists, and nine radiotherapists].

### Participant recruitment and data collection

All cases of cervical cancer meeting the eligibility criteria of an incident diagnosis were invited to participate. Women either presenting with profound hearing loss at baseline assessment, having received previous cisplatin chemotherapy, or a history of tuberculosis and/or malaria, were excluded. Prospective data collection on ototoxicity monitoring took place over a two-year period followed by a holistic, detailed reflection of the monitoring process within this institutional context. The healthcare personnel within the Oncology Department were also invited to participate in this phase of the study.

Acceptability of the ototoxicity monitoring programme was determined through the completion of questionnaires by healthcare personnel and patients at the end of the study and follow-up period, respectively. The questionnaire aimed to solicit information on the participants’ experiences and the operational processes involved in the ototoxicity monitoring programme. The questionnaire was developed taking into consideration the ASHA [14], AAA [22] and HPCSA [15] guidelines for the management of patients receiving ototoxic medication, which delineated the roles of each of the key stakeholders in an ototoxicity monitoring programme. Literature [7–9, 12, 16](highly cited articles in the field of ototoxicity) was also used to inform us about key variable selection. The questionnaire was constructed in English, translated into isiZulu and back-translated into English to ensure consistency. The questionnaire was also piloted prior to the study. Modifications were thereafter made, and any ambiguities or inconsistencies were addressed. A working definition of feasibility in the current study involved a consideration of acceptability (costs to patient and service provider) and appropriateness (through infrastructure and logistical requirements). Researcher field notes, obtained via direct observation (written immediately after leaving the field) and inference (reflecting social relationships, emotions, and meanings), as described by Neuman [26], were also used to provide insight. Potential implicit and explicit bias resulting from this process were managed using the methodology of Deggs and Hernandez [27] to ensure that the research setting was not misunderstood, misinterpreted, or misconstrued. Furthermore, the use of structured field notes also facilitated a self-reflective process allowing the researcher to be constant and systematic during data collection [27]. The time taken for each audiological evaluation was determined through the use of the participant’s tracking document which also captured the duration of each audiological procedure and evaluation.

Ototoxicity monitoring included the following audiological procedures which were conducted at all audiological evaluations namely baseline, after three-cycles of cisplatin chemotherapy, and at one-, three- and six-month post treatment: case history interview, otoscopic examination, immittance audiometry (tympanometry and acoustic reflex threshold testing), pure tone audiometry (conventional and extended high frequency range), speech audiometry (speech reception threshold testing and word recognition score testing) and Distortion product otoacoustic emission (DPOAE). All audiological procedures were undertaken in accordance protocols for ototoxicity monitoring prescribed by the American Speech-Language and Hearing Association [14]. Immittance audiometry was conducted using the GSI Tympstar V2 Impedance meter, pure tone audiometry was conducted in a soundproof booth using the Madsen Astera clinical audiometer (GN Otometrics, Schaumburg, IL. USA) and the Maico otoacoustic emissions were used for the acquisition of DPOAE data [28]. For the WRS testing, the CID W-22 Auditory test word list (Supplementary file 1) was used for English-speaking patients whilst an IsiZulu wordlist (Supplementary file 2), collated in the Discipline of Audiology, was used for IsiZulu-speaking patients. The audiometric soundproof booth and the twin channel audiometer, as described for pure tone audiometry, were used.

Patient costs were estimated based on direct non-medical costs such as transportation and income loss (employed) for the day. Loss of income was estimated using the official minimum wage categories reflected in the National Minimum Wage Act in the South African Government Gazette [29]. Transport costs (US$7.69) were based on public transport estimates for a ‘home-to-hospital’ return journey within a 50 km radius. Cumulative costs were calculated over an 8-h day for the total number of visits (5) to the health facility.

Direct service provider costs were based on the minimal audiological equipment and human resource requirements. Equipment costs were calculated from quotations received from the three major suppliers of audiological equipment in South Africa. The human resources estimate was based on the annual remuneration cost of an entry-level audiologist. Cost information (in South African Currency) was converted to $US using an exchange rate: US$1 = ZAR13.

### Data analysis

Descriptive statistics for the quantitative analysis of the questionnaires was conducted using the STATA 15 software (StataCorp. 2017, Texas, USA). Clinical analysis of all audiological test results was conducted using normative data as outlined below:

- Otoscopic examination: The different structures of the ear i.e. the pinna, ear canal, tympanic membrane and surrounding structures were recorded as normal or abnormal (pinna—presenting with sores; ear canal- reddened, or presenting with sores, discharge, foreign bodies, excessive wax, impacted wax, or blood in the ear canal; tympanic membrane—retracted, perforated, or reddened; surrounding structures- pre auricular swelling and/or swollen mastoid).
- Immittance audiometry: Tympanometric tracings were compared to Jerger (1970) classification. Acoustic reflex threshold testing measurements were based on normative data for the contralateral (70dBSL – 95dBSL) as suggested by Metz, 1952, as cited in Feldman (1978) [28] and ipsilateral (3- 6 dB SL better than contralateral) as suggested by Moller (1962) and Fria et al., (1975), as cited in Northern and Grimes (1978) [30]. An absent, reduced or elevated acoustic reflex threshold, based on the normative data was considered an abnormal result.
- Pure Tone Audiometry: The degree of hearing loss was determined using the Silman and Silverman’s (1991) [31] magnitude of hearing impairment. In addition, categories depicting the change in degree of hearing loss at frequency ranges namely mild-moderate, mild to moderately severe, mild to severe, mild to profound, moderate to moderately severe, moderate to severe, moderate to profound, moderately severe to severe, moderately severe to profound and severe to profound, were also included. Extended high-frequency audiometry thresholds were not used to classify the degree of hearing loss, as these have not been considered in the various classification systems due to the lack of consensus around normative data for this frequency range.
- Speech audiometry: A difference of 0-10 dB between the speech reception threshold and the pure tone average confirmed the validity of the pure tone results. Word recognition scores were descriptively analysed using Hodgson (1980) [32] as a guideline and categorized into excellent, good, fair, poor, very poor and extremely poor [33]. It was further categorized into a nominal variable excellent/good (1) and fair/poor/very poor/extremely poor (0).
- DPOAE results were classified as normal (1) or abnormal (0). An abnormal result was obtained when four of the six frequencies tested were found to be reduced or absent. Measurements were based on a difference between the DPOAE and the individual’s noise floor in the frequency range of 500 Hz and 8 000 Hz. An emission was considered present if the difference was equal to or greater than 6 dB and the absolute amplitude greater than -10 dB SPL [34]. A DPOAE with a reduced amplitude is one where the difference between the noise floor and the DPOAE was greater than 6 dB but the absolute amplitude was less than -10 dB SPL.

Pure tone audiometry was used to determine the NCI-CTCAE grade of ototoxicity [35], with each participant’s baseline assessment results serving as the control for each participant. Sensitivity and specificity of DPOAE and WRS were calculated using data at the 6-month post treatment follow-up with pure tone audiometry for the same period as the gold standard. Data analysis is per ear and not per patient to prevent the effects of correlation between the ears, as ototoxic hearing loss can also be unilateral, and a higher pure tone threshold in the right ear may affect the threshold in the left ear and vice versa. Qualitative data analysis was conducted using QSR International's NVIVO 12 software (Melbourne, Australia). All researcher field notes were transcribed and analysed using deductive thematic analysis [36] to yield key considerations for making the programme locally responsive.

## Results

The median age of the patient cohort was 52 years (range 32–79 years). Thirty-seven (45.1%) participants presented with stage IIB, 29 (35.4%) presented with stage IIIB cervical cancer, whereas stage IA, IB, and IIIA were less common (less than 4% each). Sixty-eight participants (82.9%) presented with co-morbidities, of which 44 (64.7%) were HIV positive and on ARTs. Patients had varied sources of income, viz. old age grants (26%), domestic workers (45%) and the remaining being unemployed (29%).

### Acceptability

Patient feedback on key aspects relating to their experience in a monitoring programme is provided in Table 1. It was reported that a large proportion (58.8%) of patients were informed about the effects of chemotherapy medication prior to treatment and that clinicians and audiologists were the main providers of such information and made the necessary referrals for audiological evaluation, respectively. Whilst 52.5% of the patients indicated that the battery of tests were time-consuming, the majority of patients followed through on the recommendations of the audiologist, the most common of which included counselling (57.5%) or referral for a hearing aid evaluation and tinnitus management (23.8%). Seventy-one participants (89%) reported having benefitted from the monitoring programme.

**Table 1:** Patient experience of the ototoxicity monitoring programme

Questions	Responses – n (%)
1. Did you receive information about the ototoxic effects of the chemotherapy medication before commencing with treatment?	No – 33 (41.3)
	Yes – 47 (58.8)
2. Who provided you with the information?	Nurse – 3 (6.4%)
	Oncologist /clinician– 36 (76.6)
	Audiologist – 7 (14.9)
	Pharmacist – 0
	Radiotherapist – 1 (2.1)
3. Who referred you for ototoxicity monitoring?	Nurse – 3 (3.8)
	Oncologist/clinician – 32 (40.0)
	Pharmacist – 1 (1.3) Audiologist – 40 (50)
	Radiotherapist – 4 (5)
4. Were the audiological evaluations conducted on the same day as your chemotherapy?	No – 53 (66.3)
	Yes – 6 (7.5)
	Sometimes – 21 (26.3)
5. Do you feel that the duration of the audiological testing was too long?	No – 38 (47.5)
	Yes – 42 (52.5)
6. Were the results of the audiological evaluations clearly explained to you?	No - 0 (0)
	Yes – 80 (100.0)
7. Which of the following recommendations were made?	Hearing aid evaluation – 3 (3.8)
	Counselling – 46 (57.5)
	Tinnitus management – 3 (3.8)
	Combination of all - 5 (6.3)
	Hearing aid evaluation and counselling – 4 (5.0)
	Hearing aid evaluation and tinnitus management – 19 (23.8)
8. Did you follow-up on any of the above recommendations made?	No -1 (1.3)
	Yes – 76 (95.0)
	Sometimes – 3 (3.8)
9. Do you feel that the monitoring of your hearing during chemotherapy was beneficial	No – 3 (3.8)
	Yes – 71 (88.8)
	Do not know – 6 (7.5)

Healthcare worker responses revealed that clinicians (50%), oncology nurses (62.5%) and radiotherapists (88.9%) treated between 70 to 100 cervical cancer patients during this two-year study period (Table 2). As per the responses received, oncology nurses (75%) and clinicains (67%) were found to be the providers of information on the ototoxic effects of medication. Enquiring about a patient’s hearing history did not emerge as common practice, with only one clinician (16.7%) and one nurse (12.9%) reporting positively. Despite more than 90% of the healthcare personnel acknowledging the benefits of an ototoxicity monitoring programme, there were varied responses to a team approach in patient management as most healthcare personnel referred less than 20 patients to the audiologist in the previous year.

**Table 2:** Healthcare personnel feedback on the operational processes of the ototoxicity monitoring programme

Question	Options	Responses per Profession n (%)			
		Clinicians (*n* = 6)	Oncology Nurses (*n* = 8)	Pharmacists (*n* = 9)	Radiotherapists (*n* = 9)
1. How many patients with cervical cancer did you treat/dispense cisplatin medication to, during the study period?	< 20	0	1 (12.5)	4 (44.4)	0
	20–50	2 (33.3)	2 (25.0)	2 (22.2)	1 (11.1)
	50–70	1 (16.7)	0	3 (33.3)	0
	70–100	3 (50.0)	5 (62.5)	0	8 (88.9)
2. Did you notice any changes in the patient’s hearing?	No	2 (33.3)	3(37.5)	9 (100)	7 (77.8)
	Yes	1 (16.7)	1(12.5)	0(0)	1 (11.1)
	Sometimes	3 (50.0)	4 (50.0)	0(0)	1 (11.1)
3. Do you provide patients with information regarding the ototoxic effects of medication?	No	1 (16.7)	2 (25)	9(42.9)	7(77.8)
	Yes	4(66.7)	6 (75)	0(0)	1(11.1)
	Sometimes	1 (16.7)	0 (0)	0(0)	1(11.1)
4. Do you provide patients with any information regarding their hearing?	No	1 (16.7)	2 (25.0)	9 (100.0)	7 (77.8)
	Yes	2 (33.3)	3 (37.5)	0 (0)	1 (11.1)
	Sometimes	3 (50.0)	3 (37.5)	0 (0)	1 (11.1)
5. Do you enquire about patient’s history of hearing difficulties?	No	0 (0)	3 (37.5)	9 (100)	6 (66.7)
	Yes	1 (16.7)	1(12.5)	0 (0)	1 (11.1)
	Sometimes	5 (83.3)	4 (50.0)	0 (0)	2 (22.2)
6. How many referrals have you made to the audiologist over the last year?	< 20	6 (100.0)	6 (75.0)	9 (100.0)	9 (100.0)
	20–50	0 (0)	1 (12.5)	0 (0)	0 (0)
	51–70	0 (0)	1 (12.5)	0 (0)	0 (0)
7. Do you feel that the ototoxicity monitoring program is beneficial?	No	0 (0.0)	0 (0.0)	2 (22.2)	1 (11.1)
	Yes	6 (100.0)	8 (100.0)	7 (77.8)	8 (88.9)
8. Was the team approach to managing the patient with cervical cancer successful?	No	1 (16.7)	0 (0)	7 (77.8)	1 (11.1)
	Yes	2 (33.3)	6 (75.0)	0 (0.0)	3 (33.3)
	Sometimes	3 (50.0)	2 (25.0)	2 (22.3)	5 (55.6)

A review of the researcher field notes further corroborated evidence that both patients and healthcare personnel predominantly displayed a positive attitude towards ototoxicity monitoring. The field notes also assisted in identifying key aspects to making the programme locally responsive; thereby, enhancing acceptability and appropriateness within the affected cohort (Table 3). Patients were found to take greater responsibility for their hearing health by actively participating in the programme, adhering to follow up appointments and being more forthcoming about their symptoms and other risk factors for ototoxic hearing loss. In the event where follow-up appointments could not be kept, patients even took initiative of rescheduling on their own accord. The interactional processes amongst health care personnel revealed a need for their roles within the programme to be well-defined and providing written feedback from the audiologist to the referring source encouraged a collaborative team approach.

**Table 3:** Key Patient and Healthcare Personnel considerations for a locally responsive ototoxicity monitoring programme

**Patient Considerations**
• Synchronizing audiology appointments with other medical appointments to the same facility
• Communication in indigenous language (isiZulu)
• Pre-treatment counselling for educating, informing and creating awareness on hearing loss and the value of early intervention; and for quelling uncertainties/fears associated with audiological procedures
• Comfort breaks during audiological testing
• Efficient referral network for other clinical/supportive care, e.g. ENT, psychologist
**Healthcare Personnel Considerations**
• Information sharing session where the roles of healthcare personnel within the programme are clearly delineated
• Communication to patients in local indigenous language (isiZulu)
• Cohesive team approach for patient referrals for baseline and follow-up audiological assessments
• Provision of written feedback to clinicians in respect of patients’ audiological assessment results, promoting a collaborative relationship between the Departments of Oncology and Audiology
• Ototoxicity information to patients made patients more responsive and increased compliance in programme

#### Time

**Fig. 1:**
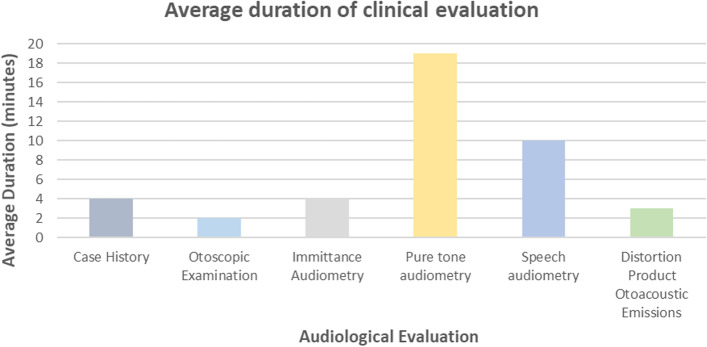
Average duration of each audiological procedure

On average, eight patients were monitored weekly. With the average audiological evaluation time of 42 min (Range: 39—45), one 8-h workday can be allocated to the ototoxicity monitoring programme for patients with cervical cancer in the current setting. As reflected in Fig. 1, pure tone audiometry, encompassing extended high-frequency audiometry was of the longest duration (19 min), followed by speech audiometry (10 min), while DPOAE testing, immittance audiometry and case history interviews ranged between 3 – 4 min.

#### Costs incurred in the implementation of- and attendance to- an ototoxicity monitoring programme

The approximate costs of equipment required for an ototoxicity monitoring programme was US$ 56 700 (Table 4). The additional expense that would be incurred by the service provider would entail the annual salary cost of an audiologist for the sole purpose of ototoxicity monitoring based on the 2021 remuneration scale of the South African National Department of Health (US$ 26 250).

**Table 4:** Costs of equipment required for ototoxicity monitoring

Equipment	Cost Estimate (US$)
Otoscope	200
Immittance meter	8 800
Pure tone audiometer with high-frequency capability	9 500
Diagnostic OAE	21 000
Soundproof booth	17 200
**TOTAL**	**56 700**

Costs incurred for unemployed patients and those receiving old age grants approximated US$38, as reflected in Table 5 whilst patients in employment incurred costs approximating US$ 98, due to loss of daily income and transport costs.

**Table 5:** Estimates of patient-related costs for attendance to the ototoxicity monitoring programme (five visits)

	**Income loss per patient [Minimum wage per hour(US$1.5) × 8 h × 5 visits]**	**Transport per patient (US$ 7.60 per visit)**	**Total per patient**
Unemployed	-	US$ 38	**US$ 38**
Old age grant recipient	-	US$ 38	**US$ 38**
Domestic worker/Day labourer	US$60	US$ 38	**US$ 98**

### Appropriateness

#### Infrastructure considerations

Responses from both patients and healthcare personnel aided in formulating a set of key considerations pertaining to infrastructure, related to the monitoring programme (Table 6). Accessibility to the healthcare facility/monitoring programme was a key aspect for patients. In addition, patients also expressed the need for a ‘fit-for-purpose’ environment, given the emotional stress from their treatment and related side effects of chemotherapy. From an audiological perspective, infrastructure designated for the programme and well-functioning, calibrated instruments were key aspects.

**Table 6:** Infrastructure considerations for a locally responsive ototoxicity monitoring programme

• The programme should be accessible by public transport
• Free/Subsidised hospital transport from referring hospitals
• Additional use of golf-carts for internal transport of patients
• A quiet, comforting waiting area
• Close proximity of ablution facilities especially for patients experiencing nausea and gastrointestinal disturbances
• A designated soundproof booth for ototoxicity monitoring
• A sound proof booth large enough for accommodating patients in wheelchairs
• Necessary audiological test equipment in good working order, with annual calibration records

#### Ototoxicity monitoring protocol assessment

Significant ototoxic change, as defined by ASHA [14] and AAA [22], was identified in all participants (100%) at the mid-cycle follow-up evaluation. The NCI-CTCAE grading criteria for assessing the adverse effect on hearing ability [35] revealed a shift in severity among 67 (83.8%) participants at the end of the 6-month post treatment evaluation, with a Grade 1 change being the most prevalent (*n* = 47, 58.8%). The comparison of test characteristics revealed that oto-acoustic emissions and word recognition score for right and left ears, respectively, presented with a high specificity (range 77.78% – 100%), whilst the sensitivity was extremely poor (range 1.45% – 22.39%) (Table 7).

**Table 7:** Comparison of audiological assessments and test characteristics in ototoxicity monitoring

**AUDIOLOGICAL ASSESSMENTS**	**Mid-cycle**	**1-month post-treatment**	**3-month post-treatment**	**6-month post-treatment**
Ototoxic hearing loss (ASHA)—n (%)	80 (100)			
NCI-CTCAE grading scale—n (%)				
*No Change*	77 (96.2)	50 (62.5)	30 (37.5)	13 (16.3)
*Grade 1*	3 (3.8)	25 (32.3)	39 (48.8)	47 (58.8)
*Grade 2*	0 (0)	4 (5.0)	5 (6.3)	9 (11.3)
*Grade 3*	0 (0)	1 (1.3)	6 (7.5)	11 (13.8)
**TEST CHARACTERISTICS**	**Sensitivity**^a^		**Specificity**^a^	
Oto-acoustic Emissions – Right	26.09%		77.78%	
Left	22.39%		81.82%	
Word Recognition Score – Right	1.45%		100%	
Left	2.99%		90.91%	

^a^ Comparisons were undertaken, using pure tone audiometry as the gold standard to detect ototoxic change at 6-month post treatment

Pure tone audiometry (reflected in Table 8) revealed sensorineural hearing loss to be most common at each audiological evaluation with mild hearing loss being the most common in the left ear, and mild-to-moderate hearing loss being the most common in the right ear at each audiological assessment.

**Table 8:** Results of Pure Tone Audiometry

	Baseline	Mid-cycle	One month post treatment	Three-month post treatment	Six-month post treatment
**Number of participants presenting with hearing loss, n (%)**					
Both ears	17(20.7)	21 (25.6)	24 (29.2)	24 (29.2)	31 (37.8)
Left ear only	6 (7.3)	7 (8.5)	4 (4.9)	10 (12.2)	10 (12.2)
Right ear only	5 (6.1)	6 (7.3)	6 (7.3)	5 (6.1)	3 (3.7)
**Degree of Hearing Loss, n (%)**					
Right ear					
Normal	60 (73.2)	55 (67.1)	51 (62.2)	51 (63.8)	46 (56.1)
Mild	8 (9.8)	8 (9.8)	11 (13.4)	8 (9.8)	11 (13.4)
Mild-to-moderate	7 (8.5)	9 (11.0)	9 (11.0)	12 (14.6)	13 (15.9)
Mild-to- moderately severe	5 (6.1)	7 (8.5)	5 (6.1)	6 (7.3)	6 (7.3)
Mild-to-severe	1 (1.2)	0 (0)	1 (1.2)	1 (1.3)	1 (1.3)
Mild-to-profound	1 (1.2)	0 (0)	1 (1.2)	1 (1.3)	1 (1.3)
Moderate-to-moderately severe	0 (0)	0 (0)	0 (0)	1 (1.3)	1 (1.3)
Moderate-to- profound	0 (0)	1 (1.2)	1 (1.2)	1 (1.3)	1 (1.3)
Left ear					
Normal	59 (72)	54 (65.9)	53 (64.6)	46 (57.5)	41 (51.3)
Mild	8 (9.8)	11 (13.4)	10 (12.2)	17 (20.7)	16 (19.5)
Mild-to-moderate	6 (7.3)	8 (9.8)	9 (11.0)	7 (8.6)	8 (9.8)
Mild-to-moderately severe	3 (3.7)	4 (4.9)	4 (4.9)	6 (7.3)	9 (11.0)
Mild-to-severe	3 (3.7)	3 (3.7)	3 (3.7)	3 (3.7)	3 (3.7)
Moderate	1 (1.2)	0 (0)	0 (0)	0 (0)	0 (0)
Moderate-to-moderately severe	0 (0)	0 (0)	0 (0)	0 (0)	2 (2.4)
Severe-to-profound	2 (2.4)	2 (2.4)	1 (1.2)	1 (1.2)	1 (1.2)
**Types of Hearing Loss**					
Sensorineural					
Right ear	21 (25.6)	25 (30.5)	28 (34.1)	28 (34.2)	33 (40.2)
Left ear	22 (26.8)	27 (32.9)	27 (32.9)	34 (41.5)	39 (47.6)
Mixed					
Right ear	1 (1.2)	2 (2.4)	1 (1.2)	1 (1.2)	1 (1.2)
Left ear	1 (1.2)	1 (1.2)	0 (0)	0 (0)	0 (0)

## Discussion

Our study is the first to inform the implementation of a locally responsive ototoxicity monitoring programme through the assessment of acceptability and appropriateness. The programme was well-received, as most participants (89%) reported it as being beneficial. The programme created greater awareness amongst healthcare personnel resulting in a larger number of referrals to the audiologist. However, informing patients about the ototoxic effects of medication, or enquiring about their hearing difficulties was not prioritized, as reflected by both healthcare personnel and patient reports. These findings further concur with Khoza-Shangase and Masondo [21] as well as Al-Malky [18], who similarly reported that referrals were only made once a patient complained of a hearing loss. Possible reasons for the lack of co-operation of all health care personnel may include large caseloads and hectic schedules [21].

A multidisciplinary team approach is considered most effective for the success of an ototoxicity monitoring programme, and the effectiveness of this approach is generally determined by the dedication and cohesivity of the team members. While most healthcare personnel displayed a positive attitude towards monitoring of a patient’s hearing, nurses were initially reluctant due to the invisible nature of a hearing loss and the fear of an increased workload. This reluctance was however minimized and further circumvented by encouraging a collaborative, cohesive and interconnected relationship. The key to developing effective collaborative partnerships is that both partners have common fundamental knowledge and skills and share a common philosophy regarding the outcome of their services [37]. While the in-service training may have improved the healthcare personnels’ knowledge regarding ototoxicity monitoring, their direct observations of the benefit of such a programme assisted in changing their attitudes. Audiologists should be the principal team member, leading efforts to implement an ototoxicity monitoring programme through the education of healthcare personnel who manage cancer patients receiving ototoxic medication, not only on the auditory effects but more especially its impact on a patient’s quality of life [16]. Highlighting the need, within a burden of disease perspective and the societal value of such a programme is key to acceptance from all relevant stakeholders to ensure a successful implementation and maintenance of an ototoxicity monitoring programme [20].

Buy-in from patients with cancer requires consideration of their context in terms of time and financial implications. A full audiological evaluation for ototoxicity in the current study lasted approximately 42 min. Despite more than half the patient cohort reporting the duration of the audiological testing being too long, they all attended follow-up visits. This compliance with the ototoxicity monitoring programme can be attributed to appropriate referrals [13] and counselling provided at all audiological evaluations. In addition, compliance with audiological recommendations were observed, highlighting that patients were taking responsibility for their hearing and that a true partnership was evolving in respect of their hearing health. This was further demonstrated by patients rescheduling audiological appointments that they could not honour due to employment commitments.

Incorporating the duration of the audiological evaluation and the process of obtaining their medical files at the hospital administration meant that most patients were unable to go to work thereafter, resulting in a loss of income for the day. With forty-five percent of the patients in this study being domestic workers and earning a minimum wage, a day away from work coupled with transport costs, can be challenging. Transport costs may be more problematic for unemployed patients or those who run their households utilising only their old age grants. In a poor household (where funds have to be rationed), funds for transport can be used to purchase essential household items such as food. This may result in patients prioritizing which appointments to attend and may opt to miss their audiology appointments for their oncology appointments. Therefore, scheduling audiological evaluations on the same day as chemotherapy visits or other medical appointments was found to be a more feasible and acceptable approach by patients, due to the savings on transport costs or not having to be away from work unnecessarily; however, this is not without limitations, which include patient fatigue, and delays in attending appointments.

Baseline testing may be influenced as patients with late-stage cancer can receive immediate treatment for their cancers, and waiting for a hearing test in the early stages of treatment may not be possible. Having an audiologist and a soundproof booth dedicated to ototoxicity monitoring within the hospital may reduce this challenge, as seen in the current study. The cost of setting up such a clinic is extremely high with the employment of an audiologist and the purchase and annual calibration of the necessary equipment exceeding US$82 000 but pays great dividends in that it allows for the early identification of ototoxicity and subsequent intervention [14]; thereby ensuring holistic management and further preventing a reduced quality of life for the ailing patient. This is especially important if one considers the current burden of disease in South Africa and its potential to increase the incidence of ototoxic hearing loss.

Inconsistent referrals to the ototoxicity monitoring programme may be addressed through an alternate method of identifying patients commencing with cisplatin chemotherapy, as baseline assessments should occur before or within 24 h of the first dose of treatment [18]. A suggestion proposed to address this challenge includes the participation of audiologists participating in oncology multidisciplinary team clinics [13]. Referrals from pharmacists in the current context were not deemed feasible due to the use of paper-based systems, making the identification of at-risk patients difficult. Therefore, in such circumstances, liaison with the radiotherapists assisted in the identification of patients not initially referred for baseline assessments, as patients with cervical cancer receive concomitant daily radiotherapy, which commences before the administration of the cisplatin chemotherapy. A transition to electronic medical records [38] may be a way to improve referrals as it would alert the pharmacist when an ototoxic medication has been prescribed, or when a patient is receiving multiple such medication. Furthermore, this study being within a public sector hospital meant a greater reliance on extensive ward stock that are not individually labelled for specific patients [38], thus increasing the difficulty of identifying patients receiving ototoxic medication. Oncology nursing staff were also helpful in this regard as they are often aware of patients receiving cisplatin medication and the approximate start date of treatment [12].

Another limitation related to scheduling the audiological evaluation on the same day as other medical appointments are the time constraints because patients may not always have the flexibility to wait for the audiological evaluation. Therefore, allocating a specific time slot for each patient may be more feasible. Additionally, selecting the most appropriate tests for the audiological evaluation may also reduce the test time. Extended high-frequency audiometry is undoubtedly the most critical audiological test that should not be omitted, as this frequency range is the most sensitive for the early detection of hearing loss [14, 22]. This consequently led to the development of the sensitive region for ototoxicity (SRO) [39], which may assist in reducing patient test time. The SRO is “the highest frequency with a threshold at or below 100 dB Sound Pressure Level (SPL) followed by the next six lower adjacent frequencies in 1/6-octave steps or the one-octave range near the highest audible frequency” [40]. With 94% sensitivity in detecting ototoxic change, whether in the conventional [41] or extended frequency range [42], the SRO is a valuable tool. Despite its benefit of a rapid test time, the SRO was not included in the current study, as it has been omitted from ASHA, AAA, and HPCSA protocols probably due to a lack of equipment availability [39].

Another audiological test considered excellent for ototoxic monitoring is DPOAE testing. It is noted for its objective, frequency-specific nature, quick administration, and ability to reveal subclinical cochlear damage before pure tone thresholds are affected [16]. In the current study DPOAEs were not sensitive in detecting significant ASHA ototoxic change. The current authors postulate that these results may be due to most participants in the study presenting with normal hearing or mild to moderate degrees of hearing loss in the conventional frequency range, and DPOAEs are not as sensitive to slight or mild hearing losses [43]. Furthermore, the DPOAE measurement was possibly limited to the conventional frequency range, while a significant ototoxic change in the current study was most evident in frequencies above 8000 Hz, and the DPOAE equipment used in the current study could assess up to 6000 Hz. Therefore, it is advisable to ensure that DPOAE equipment utilised for ototoxicity monitoring has the capability of assessing at least up to 10 000 Hz, as higher DPOAE frequencies have been reported to be statistically more sensitive to early ototoxicity than lower DPOAE frequencies [44].

Whilst DPOAE testing is encouraged at all audiological evaluations, speech audiometry (incorporating speech reception threshold and word recognition score) is conducted at the baseline assessment and when there is a significant change in air conduction thresholds [16]. The sensitivity of word recognition score testing in detecting a significant ototoxic change in the current study reflects that this test does not significantly contribute to the diagnosis of ototoxic hearing loss. A possible postulation about why word recognition scores are a poor indicator of ototoxicity includes the use of monitored live voice testing instead of recorded speech material. Despite the many disadvantages of monitored live voice testing [45], this method of presentation was utilized due to the lack of the necessary equipment at the study site, a common issue affecting many institutions in limited resource environments. Furthermore, with the results of speech audiometry often being used to counsel patients about the impact of hearing loss on communication [16], it is imperative that speech audiometry is conducted in noisy and quiet conditions [8]. While speech in noises tests are not commonly utilized during ototoxicity monitoring [14, 22], its inclusion in the test battery would be beneficial in determining or predicting real world difficulties likely to be experienced by patients with cancer and would therefore assist in planning appropriate rehabilitation [8]. In the current study, speech audiometry was conducted at all audiological evaluations and added approximately 10 min to the session. Considering the poor sensitivity and specificity of speech (in quiet) audiometry, the HPCSA [15] has not recommended its inclusion in the monitoring audiometry. Therefore, the current authors recommend the investigation of the inclusion of speech in noise testing during ototoxicity monitoring, given the reasons highlighted above.

However, HPCSA [15] recommends bilateral otoscopic examination, bilateral pure-tone air conduction testing, and bilateral DPOAEs conducted bi-weekly during treatment. Conducting the full audiological test battery at each audiological evaluation is time-consuming, as reported by most participants, highlighting the need to modify the test battery [16]. Konrad-Martin et al. [13] also revealed that multiple comprehensive audiological evaluations were not feasible for monitoring assessments, even for responsive patients. Therefore, a more workable option may be a weekly evaluation prior to receiving cisplatin treatment, as suggested by ASHA [14] and AAA [22] so that significant ototoxic change is detected to allow early treatment modifications within the ambit of practice guidelines and appropriate audiological interventions.

Whilst all patients were identified with ototoxicity at their mid-cycle follow-up, further assessment using the NCI-CTCAE criteria aided in the grading of severity. This approach, in line with the HPCSA [15], allowed for an evidence-informed approach to aural rehabilitation to mitigate the functional impact that the degree of hearing loss can have on a patient’s communication [16]. The need for coherently and meaningfully communicating a patient’s audiological assessment results to the necessary stakeholders was key in ensuring early patient-specific therapeutic interventions [46]. In addition, providing feedback to patients with minimal jargon also encouraged compliance to the recommendations made.

In the current study, the strategy of ‘creative scheduling’ (during lunch breaks and before the audiology clinic official hours) and having at least two blank appointments each day were adopted to accommodate for baseline assessments or patients who were delayed for their appointments or had many appointments on a day. This plan was suitable for the current study due to patients with cervical cancer only being assessed; however, it may not work for an ototoxicity monitoring programme that caters for all patients receiving cisplatin chemotherapy at the facility. Therefore, it is advisable for the ototoxicity monitoring programme to be set up in a location close to the chemotherapy treatment room, or use portable equipment such as that used in teleaudiology, namely the KuduWave [47] or OtoID [48], which may address some of the challenges of the conventional audiological ototoxicity monitoring programme.

This study, while having the advantage of reporting on real world practice, is not without limitations. Being a single site study, the findings of this study have limited generalizability, perhaps to similar environments looking to initiate such programmes and highlights the need for more engagement on this topic. Further studies, exploring key attributes to enhance the success of such programmes, highlight the barriers, gaps and local needs not identified/ investigated in this study, are required to build on the evidence base. This can be achieved through further qualitative research methodologies such as one-on-one and focus group interviews, which would allow for data enrichment and data saturation. This would also promote robust dialogue and encourage a sharing of experiences on best practices within similar resource settings, resulting in greater mobility for alignment with the recommendations of the World Health Organization [49] of making ear and hearing care part of universal health coverage.

## Conclusion

This study is the first to comment on the feasibility of ototoxicity monitoring in South Africa that has reflected the realities of our community and practice setting. It provides insight for a contextually responsive programme within the scope of the HPCSA guidelines and can serve as a potential roadmap for other limited resource environments considering the implementation, adaptation, integration or expansion of such a program. The study revealed that an ototoxicity monitoring programme is feasible through meaningful dialogues and partnerships with- and considerations from- both patients and health care providers regarding planning, delineation of responsibilities and cost implications. Whilst the need for protocol refinement is another area that requires a greater depth of discussions, it is abundantly clear that an established and well-functioning ototoxicity monitoring programme allows for a proactive hearing health promotion culture, allowing for the early identification of ototoxic hearing loss and subsequent rehabilitation of the unavoidable hearing loss, and its benefits therefore outweigh its costs.

## Supplementary Information

**Additional file 1.** CID W22 auditory test wordlist for word recognition score testing for english speaking participants. 

**Additional file 2.** Word recognition score wordlist for isizulu speaking participants.

## Data Availability

The datasets used and/or analysed during the current study available from the corresponding author on reasonable request.

## Abbreviations

AAA: American Academy of Audiology
ASHA: American Speech-Language-Hearing Association
DPOAE: Distortion product otoacoustic emission
HPCSA: Health Professions Council of South Africa
HIV: Human Immunodeficiency Virus
LMIC: Low-Middle Income countries
SRT: Speech recognition threshold
WRS: Word recognition score/ speech discrimination
