# Supplementary material for: Cisplatin-associated ototoxicity: perspectives from a single institution cervical cancer cohort and implications for developing a locally responsive monitoring programme in a public healthcare setting
Source: BMC Health Serv Res. 2022 Jun 18;22:791. doi: 10.1186/s12913-022-08099-8 (PMC9206275; doi:10.1186/s12913-022-08099-8)
Supplement: Supplementary file 1 — Additional file 1. CID W22 auditory test wordlist for word recognition score testing for english speaking participants. [file 12913_2022_8099_MOESM1_ESM.docx]

**Cisplatin-associated Ototoxicity: Perspectives from a single institution cervical cancer cohort and implications for developing a locally responsive monitoring programme in a public healthcare setting**

Jessica Paken*, Cyril D. Govender, Mershen Pillay, Vikash Sewram*

**CID W22 AUDITORY TEST WORDLIST FOR WORD RECOGNITION SCORE TESTING FOR ENGLISH SPEAKING PARTICIPANTS**

| LIST 1 A | | LIST 2A | |
| --- | --- | --- | --- |
| An | you (ewe) | yore (your) | and |
| Yard | As | bin (been) | young |
| Carve | Wet | way (weigh) | cars |
| Us | Chew | chest | tree |
| Day | see (sea) | then | dumb |
| Toe | Deaf | ease | that |
| Felt | Them | smart | die (dye) |
| Stove | Give | gave | show |
| Hunt | True | pew | hurt |
| Ran | Aisle | ice | own |
| Knees | or (or) | odd | key |
| not (knot) | Law | knee | oak |
| Mew | Me | move | new (knew) |
| Low | none (nun) | new | live (verb) |
| Owl | Jam | jaw | off |
| It | Poor | one | ill |
| She | Him | hit | rooms |
| High | Skin | send | ham |
| there (their) | East | else | star |
| earn (urn) | Thing | tare (tear) | eat |
| Twins | Dad | does | thin |
| Could | Up | too (two, to) | flat |
| What | Bells | cap | well |
| Bathe | Wire | with | by (buy) |
| Ace | Ache | air (heir) | ail (ale) |

| List 3A | | List 4A | |
| --- | --- | --- | --- |
| Bill | Aim | all | dam |
| add (ad) | When | wood | art |
| West | Book | at | will |
| Cute | Tie | where | dust |
| Smart | Do | chin | toy |
| Ears | Hand | they | aid |
| Tan | End | dolls | than |
| Nest | Stove | so (sew) | eyes |
| Say | Have | nuts | shoe |
| Is | Owes | ought (aught) | his |
| Out | Jar | in (inn) | our |
| Lie (lye) | no (know) | net | men |
| Three | May | my | near |
| Oil | Knit | leave | few |
| King | On | of | jump |
| Pie | If | hang | pale |
| He | Raw | save | so |
| Smooth | Glove | ear | stiff |
| Farm | Ten | tea (tee) | can |
| This | Dull | cook | through |
| done (dun) | Though | tin | clothes |
| use (yews) | Chair | bread (bred) | who |
| Camp | We | why | bee |
| Wool | ate (eight) | arm | yes |
| Are | Year | yet | am |
