# Supplementary material for: Cisplatin-associated ototoxicity: perspectives from a single institution cervical cancer cohort and implications for developing a locally responsive monitoring programme in a public healthcare setting
Source: BMC Health Serv Res. 2022 Jun 18;22:791. doi: 10.1186/s12913-022-08099-8 (PMC9206275; doi:10.1186/s12913-022-08099-8)
Supplement: Supplementary file 2 — Additional file 2. Word recognition score wordlist for isizulu speaking participants. [file 12913_2022_8099_MOESM2_ESM.docx]

**Cisplatin-associated Ototoxicity: Perspectives from a single institution cervical cancer cohort and implications for developing a locally responsive monitoring programme in a public healthcare setting**

Jessica Paken*, Cyril D. Govender, Mershen Pillay, Vikash Sewram*

**WORD RECOGNITION SCORE WORDLIST FOR ISIZULU SPEAKING PARTICIPANTS**

| Sa | Mhlophe | umuthi | woza |
| --- | --- | --- | --- |
| Indlebe | Mnyama | tshela | sebenza |
| Amehlo | Luhlaza | nxa | bhala |
| Ubuso | Nsundu | mama | izwa |
| Unyawo | Mdubu | namhlanje | mntwana |
| Ikhanda | Mpunga | munca | ifasitela |
| umlenze | Bomvu | shesha | ummese |
| Umlomo | Mpofu | ncane | inkomisi |
| Ikhala | Namuhla | na | isitulo |
| Impama | Ilanga | funda | hleka |
| Ulimi | Cela | inyanga | imigwaqo |
| Inyoni | Isijula | malusi | donsa |
| Ikati | Cishe | amanzi | izitofu |
| izinkomo | Dlalisa | mnyama | hlula |
| Iqhude | Xwaya | isho | inkosi |
| Ihashi | Incwadi | umlungu | futhi |
| ivondwe | Tsheleka | umfazi | izilimi |
| ubhejane | Qhuba | isitemela | inyoka |
| Insephe | Xabana | izinyo | indlovu |
| Inqe | Wowa | fundisa | phuza |
| umhlanga | Yabo | ushukela | ubisi |
| Ihlobo | Yekela | odade | khipha |
| Ikamelo | Yini | gijima | indlu |
| ubusuku | zama-zama | izambane | umuntu |
| Shiya | Cezulu | ukudla | phezulu |
